# Iron and Cancer: A Special Issue

**DOI:** 10.3390/cancers15072097

**Published:** 2023-03-31

**Authors:** Yasumasa Okazaki, Keisuke Hino

**Affiliations:** 1 Department of Pathology and Biological Responses, Nagoya University Graduate School of Medicine, 65 Tsurumai-cho, Showa-ku, Nagoya 466-8550, Japan; 2 Department of Hepatology and Pancreatology, Kawasaki Medical School, 577 Matsushima, Kurashiki 701-0192, Japan

Iron is an essential element for all organisms, and iron-containing proteins play critical roles in cellular functions. The biological importance of iron is largely attributable to its chemical properties as a transitional metal. However, an excess of “free” reactive iron damages the macromolecular components of cells and cellular DNA through the production of harmful free radicals [1]. For instance, hepatocellular carcinoma (HCC), malignant mesothelioma, and ovarian clear-cell carcinoma have been shown to develop as a result of excess iron and/or oxidative stress caused by free radicals [2]. In the last decade, accumulating evidence regarding molecules regulating iron metabolism or iron-related cell death programs, such as ferroptosis, has shed light on the relationship between excess iron and carcinogenesis. Furthermore, iron depletion by chelating is shown to be a promising therapeutic option for osteoporosis, inflammatory bowel diseases, and several cancers, in addition to hemoglobinopathies [3]. In this Special Issue, four original articles and one review are presented.

In a non-neoplastic liver, glutamine synthetase (GS) is exclusively expressed in zone 3 hepatocytes, while in almost all β-catenin-mutated hepatocellular adenoma and 35–60% of HCC, diffuse and strong expression of GS is a hallmark of tumorigenesis [4], indicating GS expression is activated by Wnt/β-catenin signaling. Furthermore, the activation of the Wnt/β-catenin pathway induces organic anion transporting polypeptide 1B3 (OATP1B3) expression, which enables the detection of HCC as a nodule with a higher enhancement of gadolinium–ethoxybenzyl–diethylenetriamine (Gd-EOB-DTPA)-enhanced magnetic resonance imaging (MRI) [5]. The study by Hamaguchi et al. [6] reports that a relative enhancement ratio of Gd-EOB-DTPA-enhanced MRI and the expression of GS, which indicates the activation of Wnt/β-catenin signaling pathway, and 8-oxo-dG (8-oxo-2′-deoxyguanosine) were significantly associated with elevated serum ferritin levels in nonalcoholic fatty liver disease (NAFLD) patients with HCC. In NAFLD patients, hyperferritinemia is thought to be caused by iron accumulation or inflammation [7]. This article clearly demonstrates the association between hepatic iron deposition, assessed by iron staining and elevated ferritin in sera.

To date, the clinical application and use of nanomedicine in cancer therapy is still at an early stage. Biocompatible nanoparticles, which contain inorganic materials, such as iron, gold, silver, platinum, titanium, and silica, are currently being developed, among which iron nanoparticles retain a high magnetism and are applied as an alternative mapping compound by coating with carboxydextran [8]. When resecting a breast cancer completely to prevent recurrence, surgeons search for a sentinel lymph node (SLN), where cancer cells are initially seeded via the lymphatic flow, to reduce invasive damage at the axillary fossa. Currently, dyes and/or tracing radioisotopes are used to detect SLN in clinical practice. Taruno et al. [9] demonstrate that the rate of SLN identification by super paramagnetic iron oxide (SPIO) nanoparticles is equivalent to a radioisotope tracer or dye, without provoking any significant side effects, such as allergic reactions, prolonged dermopigmentation, or MRI artifacts. A subsequent systemic review and meta-analysis revealed that SPIO nanoparticles could be considered an alternative standard for detecting SLN [10], indicative of advances being made in nanomedicine-based clinical applications.

The oral intake of high-dose ascorbate (10 g/day) first began in the 1970s for patients with advanced cancers [11]. In the 2000s, the intravenous administration of ascorbate increased its plasma concentration to several hundred times higher than by oral intake [12]. Since then, high-dose ascorbate, which is pharmacological but not physiological, has been explored as a therapeutic option due to its higher selectivity to cancer cells than normal cells. Pharmacological ascorbate increases extracellular H_2_O_2_ and DNA demethylation by activating ten-eleven translocation (TET) [12] and cytoplasmic labile iron with a positive feedback loop that amplifies toxicity in cancer cells [11]. Here, Qiu et al. [13] demonstrate that high-dose ascorbate inhibited cellular proliferation in cancer cells, although sensitivity to ascorbate differed between the cell lines. Furthermore, iron supplementation was found to enhance ascorbate-induced cytotoxicity. Conversely, the knockdown of transferrin receptor 1 decreased this effect and ^68^Ga uptake. In a recent study, a T2* MRI taken 4 h after pharmacological ascorbate infusion or radiation treatment indicated that the Fe^2+^/Fe^3+^ ratio in the tumoral area of glioblastoma multiforme patients was significantly increased compared with the non-neoplastic location of cerebrum [14]. This provides promising evidence for the use of pharmacological ascorbate for iron-involved cancer therapy.

Iron is stored as chelatable iron within labile iron pool, in addition to ferritin. The chelatable iron forms hydrated complexes with low-molecular-weight chelates, such as ascorbate, ADP, citrate, or glutathione; thus, chelatable iron is susceptible to reduction from ferric (Fe^3+^) to ferrous ion (Fe^2+^) [15]. Fe^2+^ catalyzes the production of hydroxyl radicals (^•^OH) by decomposing H_2_O_2_ via the Fenton reaction. Iron-mediated ROS yields secondary oxidants and electrophiles, which may modulate redox signaling [16]. In this Special Issue, Igarashi et al. [17] demonstrate that H_2_O_2_ efficiently induced cell death in rat thymocytes, while the preincubation of FeSO_4_ was necessary to induce cytotoxicity with H_2_O_2_. The supercoiled form of plasmid DNA was efficiently disassembled by H_2_O_2_ with ultraviolet irradiation but not H_2_O_2_, with FeSO_4_ ideally located at a longer intermolecular distance between plasmid and ^•^OH, indicating that the distance between ^•^OH and biomolecules is critical for direct damage.

As mentioned above, chelatable iron, which is also known as redox-active or catalytic iron, triggers oxidative stress-mediated cellular damage that ultimately causes organ impairments, such as cancer, hemochromatosis (hepatic cirrhosis, diabetes mellitus, cardiomyopathy, and endocrine dysfunction), and ischemia/reperfusion injury in the brain, heart, and kidney [18]. Here, Okazaki [19] summarizes the current understanding of a ferric nitrilotriacetate (Fe-NTA)-induced renal carcinogenesis model. Fe-NTA, which forms a μ-oxo dimer iron at a neutral pH, initiates lipid peroxidation and cell death in the kidney and liver. Indeed, male rodents are vulnerable to Fe-NTA-induced renal injury, which is associated with ferroptosis and attenuated by castration and/or estriol administration, indicating that sex hormones are critical for the suppression of iron-dependent lipid peroxidation. When *glutathione peroxidase 4* (*Gpx4*), which maintains the central defense against lipid peroxidation, was conditionally deleted, murine female kidneys were found to be protected from ferroptosis in proximal tubule cells; indeed, male patients suffer more frequently from acute kidney injury than their female counterparts [20]. Taken together, these findings provide the basis for the development of a new therapeutic strategy to either suppress ferroptosis for alleviating damage or to induce ferroptosis for killing cancer cells.

In conclusion, this Special Issue presents two clinical studies that explore the role of iron in hepatocarcinogenesis and the diagnostic application of iron nanoparticles in breast cancer, in addition to two further studies that explore the role of iron with high-dose ascorbate and the modification of biomolecules from iron-elicited ^•^OH. Furthermore, the featured review presents an iron-induced renal carcinogenic animal model and discusses ferroptosis with the aim of understanding pathogenesis-based therapy and prophylaxis. We hope that this Special Issue will attract readers interested in iron metabolism and oxidative stress, as well as the biomedical application of iron oxide nanoparticles.
